# Can Green Innovation Improve Regional Environmental Carrying Capacity? An Empirical Analysis from China

**DOI:** 10.3390/ijerph192013034

**Published:** 2022-10-11

**Authors:** Juan Hu, Chengjin Ma, Chen Li

**Affiliations:** 1 School of Discipline Inspection and Supervision, Huanggang Normal University, Huanggang 438000, China; 2 Collaborative Innovation Center for Emissions Trading System Co-Constructed by the Province and Ministry, Wuhan 430205, China; 3 Warner College of Natural Resources, Colorado State University, Fort Collins, CO 80523, USA; 4 School of Management, Shanghai University of Engineering Science, Shanghai 201620, China

**Keywords:** green innovation, regional environmental carrying capacity, PM2.5 concentration, spatial spillover effect, China

## Abstract

Green innovation has become an important driving force for China’s economic transformation and development. This paper selects the 2010–2020 provincial-level regions in China as samples, and adopts a multi-indicator comprehensive evaluation method to comprehensively, objectively and scientifically evaluate the environmental carrying capacity of air pollution in two dimensions: natural resource endowment and human activity impact, and also measures and calculates the green innovation in each province, city and autonomous region to explore the specific impact of green innovation on environmental carrying capacity and its spatial spillover effect; it also explores the heterogeneous effects of green innovation on environmental carrying capacity under different pollution environments. The conclusions show that: (1) Green innovation has a positive impact on environmental carrying capacity. (2) There is a spatial spillover effect of green innovation on environmental carrying capacity. In other words, in areas with higher PM2.5 concentration, that is, lower environmental quality, green innovation has a weaker ability to improve environmental carrying capacity; in areas with lower PM2.5 concentration, that is higher environmental quality, green innovation has a stronger ability to improve environmental carrying capacity. (3) In the process of green innovation affecting environmental carrying capacity, PM2.5 plays the part of a mediating effect, indicating that green innovation is an intermediate transmission mechanism affecting environmental carrying capacity, and the results show that the absolute value of the short-term indirect effect is greater than the absolute value of the short-term direct effect, and the long-term direct effect is greater than the long-term indirect effect.

## 1. Introduction

The ecological environment is the foundation of human survival and development, and it is the common wish of all people from all countries to maintain a good ecological environment. In recent years, China’s ecological environment protection is still in a critical period of superimposed pressure and heavy burden; the results of ecological environment quality improvement are not solid, and ecological environment protection has a long way to go [1,2,3,4]. China’s ecological and environmental problems are essentially a matter of high-carbon energy structure and high energy consumption and high-carbon industrial structure, and pollutants and carbon dioxide emissions show significant homology. Almost all of China’s major air pollutant emission sources, including sulfur dioxide and nitrogen oxides, as well as about 50% of volatile organic compounds and 85% of primary PM2.5 emissions (excluding dust), are highly congruent with carbon dioxide emissions [5,6,7,8]. Therefore, during the key strategic period of China’s economic transition from the pursuit of scale to the pursuit of quality, it has become the consensus of the whole society to rely on science and technology innovation to solve the problem of development and environmental balance, and green innovation with energy saving and emission reduction as the key technology has become an important means of green transition development and environmental governance [9,10,11].

Green innovation is a general term for “pollution-free” or “less pollution” technologies, processes and products that follow ecological principles and ecological economic laws, save resources and energy, avoid, eliminate or reduce ecological pollution and damage, and minimize negative ecological effects. Its content mainly includes: pollution control and prevention technology, source reduction technology, waste minimization technology, recycling technology, ecological process, green products, purification technology, etc. [12,13,14,15,16,17]. It can be seen that green innovation is a new type of modern technology system in harmony with the ecological environment system. Green innovation is also called eco-technology innovation, which is a kind of technological innovation [18]. Generally, management innovation and technological innovation with the goal of protecting the environment are collectively referred to as green innovation. There are two main ways to define green innovation, starting from the characteristics of green innovation and outlining the main features to arrive at the definition, and considering the production process and making a systematic description of the green innovation process [19,20,21,22,23]. In the context of the dramatic increase in resource pressure and increasingly stringent environmental protection standards, a large number of green innovations have emerged in all fields except environmental protection and its related industries, which have had a profound impact on the macro social operation and the development of micro market players [24,25,26,27,28,29,30]. Macro themes such as green performance, green benefits and diffusion mechanisms of science and technology innovation have been attracting much attention, while empirical studies on some micro issues, such as the factors influencing green innovation of enterprises and the efficiency of green innovation of enterprises, have also accumulated a lot of practical experience on the specific operation of green innovation in China [31,32,33,34,35,36]. In addition, some scholars have also studied green management innovation independently [37,38,39,40,41,42]. At this time, green production technology innovation mainly includes the innovation of green product design, green materials, green process, green equipment, green recycling and treatment, green packaging and other technologies; green production management innovation includes the formulation of green enterprise management mechanism, green cost management innovation, the adoption of advanced production methods, the establishment of green marketing mechanism, the establishment of green networked supply chain and the establishment of environmental evaluation and management system [43,44,45,46,47,48].

Environmental carrying capacity is also called environmental tolerance or environmental endurance [49,50,51,52,53]. It refers to the limit of the ability of a region’s environment to support human social and economic activities in a certain period of time and in a certain environmental state, which reflects the maximum pollutants allowed by the environmental unit, the ability of a specific environmental unit to permanently carry the discharge of human activities [54,55,56,57,58,59,60]. Therefore, whether it is affected by green innovation or changes in different scenarios that are significantly different from other environmental indicators, the existing findings of established environmental capacity or carrying capacity studies are not directly applicable to environmental carrying capacity [61,62,63]. Green innovation and environmental carrying capacity are intrinsically linked in a complex way. As an innovation directly pursued by environmental benefits, green innovation can not only improve the environmental absorption capacity from the absorption side by transforming the ecosystem, but also improve the environmental carrying capacity of pollutants by coupling with the existing environmental absorption characteristics on the pollution emission side [64,65,66]. At present, enterprises, as an important subject of environmental protection and governance, enhance the environmental carrying capacity through green innovation indirectly, and the impact of green innovation on environmental carrying capacity is not linear in the context of China’s current environmental carrying capacity, which is generally close to the upper limit. Most studies agree that green innovation will have a significant effect on reaching energy saving and emission reduction through technological upgrading and scientific transformation, where an important mechanism is the enhancement of environmental carrying capacity.

Whether the impact of green innovation on environmental carrying capacity, an environmental indicator, meets the expectation of energy saving and emission reduction, identifying the specific environmental and economic effects of relevant green innovation, and the similarities and differences of the impact mechanisms of green innovation on environmental carrying capacity in different contexts is of great interest. Therefore, understanding the specific direction, extent and mechanism of green innovation on environmental carrying capacity and its moderating factors under heterogeneous scenarios is extremely valuable for exploring environmental carrying capacity, strengthening the effect of air pollution control and broadening the ideas of air quality improvement. Therefore, this paper takes Chinese provincial regions as the research object, adopts a multi-indicator comprehensive evaluation method, measures the green innovation capacity and environmental carrying capacity of Chinese regions from two dimensions of natural resource endowment and human activity influence, explores the specific influence of green innovation on environmental carrying capacity and its spatial spillover effect and explores the heterogeneous influence of green innovation on environmental carrying capacity under different pollution environments, so as to provide a theoretical basis and a basis for environmental governance and regional sustainable development. It also explores the heterogeneous effects of green innovation on environmental carrying capacity in different polluted environments, and provides a theoretical basis and decision-making reference for environmental governance and regional sustainable development.

## 2. Model Construction and Data Selection

### 2.1. Benchmark Model

In this paper, based on the STIRPAT model and the EKC hypothesis [67,68,69,70], the following benchmark model was constructed to examine the impact of green innovation on regional environmental carrying capacity:

(1) $$\ln{\mathrm{ECC}}_{\mathrm{it}}=\alpha_{0}+\alpha_{1}\ln{\mathrm{Gino}}_{\mathrm{it}}+\alpha_{2}{\left(\ln{\mathrm{Gino}}_{\mathrm{it}}\right)}^{2}+\alpha_{3}X_{\mathrm{it}}+\varepsilon_{\mathrm{it}}$$

In this, *i* is a cross-sectional unit of 31 provincial regions in mainland China and *t* denotes the year; *ECC* is the explanatory variable regional environmental carrying capacity, *Gino* is the core explanatory variable green innovation capacity and *X* is a set of control variables; *α*_0_–*α*_3_ are parameters to be estimated; *ε* is a random disturbance term.

### 2.2. Spatial Econometric Model

The three most common spatial econometric models for cross-sectional data include the spatial autoregressive model (SAR), the spatial error model (SEM) and the spatial Durbin model (SDM) [71,72,73]. The spatial autoregressive model (SAR) is usually applied when the dependent variable is spatially correlated over regions, the spatial error model (SEM) is applied when the independent variable is spatially correlated over regions and the spatial Durbin model (SDM) is an extended form of the spatial lag model and the spatial error model, which considers the role of autocorrelation of both the dependent variable and the independent variable, and the model contains both the independent variable and the dependent variable spatial hysteresis, considering that the dependent variable haze pollution (PM) and the main independent variable urban innovation efficiency (E) are spatially correlated in this paper. In order to ensure the reliability and scientific of the statistical results, this paper, therefore, uses the spatial Durbin model (SDM) for subsequent measurement to better estimate the spatial spillover effect of urban innovation efficiency and its influence role on haze pollution in neighboring cities.

The general form of the spatial Durbin model (SDM) is as follows:

(2) $$y=\alpha \tau_{n}+\rho W_{y}+X\beta +WX\delta +\varepsilon$$

Based on the form of the base model used above, the model after taking the natural logarithm of the indicators on both sides of the equation is:

(3) $$\ln{\mathrm{ECC}}_{\mathrm{it}}=\beta_{0}+\rho \sum_{j=1,j\neq i}^{N}w_{\mathrm{ij}}\ln{\mathrm{ECC}}_{\mathrm{it}}+X_{\mathrm{it}}\beta +\sum_{j=1,j\neq i}^{N}w_{\mathrm{ij}}X_{\mathrm{it}}\theta +\mu_{\mathrm{it}}$$

(4) $$\mu_{\mathrm{it}}=\varphi \sum_{j=1,j\neq i}^{N}w_{\mathrm{ij}}\mu_{\mathrm{it}}+\varepsilon_{\mathrm{it}}$$

Among them, *w_ij_* denotes the elements of the geographic distance spatial weight matrix W1 constructed based on the geographic distance between regions, the residual term is *μ_ij_*, *ρ* is the spatial autoregressive coefficient, *φ* is the spatial autocorrelation coefficient and *X* is the set of independent variable vectors.

### 2.3. Mediating Variable Model

In this paper, a mediating effects model consisting of the following three regression equations is constructed to test the identification of transmission pathways:

(5) $$\ln{\mathrm{ECC}}_{\mathrm{it}}=\theta_{0}+\theta_{1}\ln{\mathrm{Gino}}_{\mathrm{it}}+\theta_{2}{\left(\ln{\mathrm{Gino}}_{\mathrm{it}}\right)}^{2}+\theta_{3}Y_{\mathrm{it}}+\xi_{\mathrm{it}}$$

(6) $$D_{\mathrm{it}}=\beta_{0}+\beta_{1}\ln{\mathrm{Gino}}_{\mathrm{it}}+\beta_{2}{\left(\ln{\mathrm{Gino}}_{\mathrm{it}}\right)}^{2}+\beta_{3}Y_{\mathrm{it}}+\mu_{\mathrm{it}}$$

(7) $$\ln{\mathrm{ECC}}_{\mathrm{it}}=\gamma_{0}+\gamma_{1}\ln{\mathrm{Gino}}_{\mathrm{it}}+\gamma_{2}{\left(\ln{\mathrm{Gino}}_{\mathrm{it}}\right)}^{2}+\gamma_{3}Y_{\mathrm{it}}+\gamma_{4}D_{\mathrm{it}}+\tau_{\mathrm{it}}$$

*Y* is the vector set composed of control variables; *D* is the mediating variable PM2.5; *ECC* is the explanatory variable regional environmental carrying capacity, and *Gino* is the core explanatory variable green innovation capacity [74]. According to the principle of the mediating effect model, if the coefficients *θ*_1_ or *θ*_2_, *β*_1_ or *β*_2_, *γ*_4_ are significant, and the coefficients *γ*_1_ and *γ*_2_ become smaller or less significant than *θ*_1_ and *θ*_2_, it indicates that there is a mediating effect.

### 2.4. Research Object and Variable Selection

#### 2.4.1. Research Object

**Figure 1:**
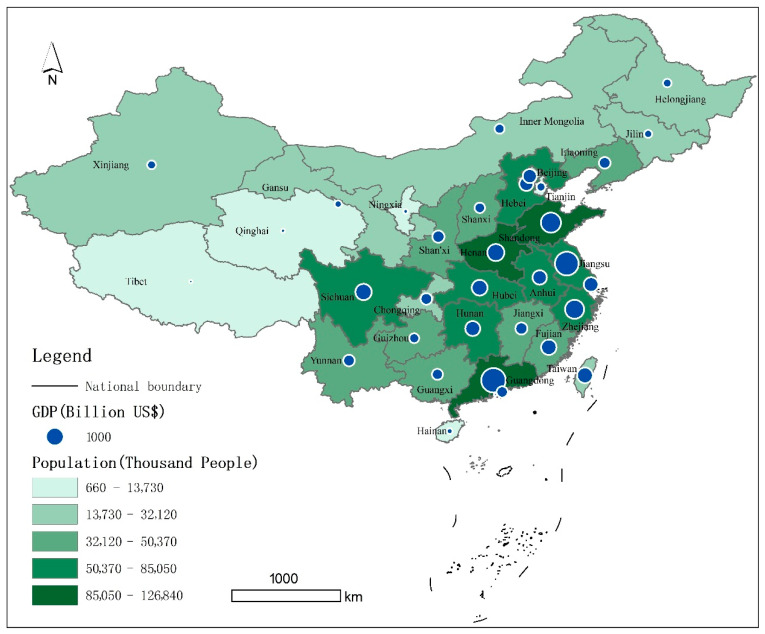
Schematic Diagram of the Study Object.

Our study covers 31 provincial administrative regions in China. Due to a lack of data, China’s Taiwan Province, Hong Kong Special Administrative Region and Macau Special Administrative Region have not yet been included in our evaluation system. At the end of 2021, there were 34 provincial administrative regions in China, including 23 provinces, 5 autonomous regions, 4 municipalities directly under the Central Government and 2 special administrative regions. Beijing is the capital of China. China is divided into four main regions, namely the Northeast Region, the Eastern Region, the Central Region and the Western Region, of which the Northeast Region includes three provincial administrative regions, namely Liaoning, Jilin and Heilongjiang; the Eastern Region includes 13 provincial administrative regions or special administrative regions, including Beijing, Tianjin, Hebei, Shanghai, Jiangsu, Zhejiang, Fujian, Shandong, Guangdong, Hainan, Taiwan, Hong Kong and Macau; the Central Region includes six provincial administrative regions, including Shanxi, Anhui Jiangxi, Henan, Hubei and Hunan, and the western region includes 12 provincial-level administrative regions, including Inner Mongolia, Guangxi, Chongqing, Sichuan, Guizhou, Yunnan, Tibet, Shaanxi, Gansu, Qinghai, Ningxia and Xinjiang. In terms of the degree of economic development, the eastern region of China is relatively developed, including economically developed regions such as Guangdong, the largest economic province, Shanghai, the largest economic city, Taiwan Province, and Hong Kong. In terms of resident population, the eastern coastal regions are notable for the size of their population, as they are both economically developed provinces and among the most densely populated regions in China. In contrast, the vast western regions of China are less densely populated and less economically dense. In 2021, the gross regional product of China’s eastern, central, western and northeast regions were USD 10,389 billion, USD3867 billion, USD 3715 billion and USD 863 billion, respectively, totaling USD 18,835 billion (including data from Taiwan, Hong Kong and Macau, China), and the size of the resident population of the four regions were 597,500 thousand, 364,450 thousand, 382,810 thousand and 97,290 thousand, respectively, totaling 1,442,050 thousand (including data from Taiwan, Hong Kong and Macau, China).The geographic and spatial distribution forms a pattern of population-economic-social development that decreases in an east-central-west gradient (Figure 1).

#### 2.4.2. Variable Selection and Data Sources

The explanatory variable is Environmental Carrying Capacity (ECC), which is measured by the comprehensive weighting method. In the process of constructing the indicator system, seven representative indicators are selected from two dimensions, namely, endowment of natural conditions and influence of human activities, by drawing on the research results of scholars from various disciplines, to systematically construct an indicator system for measuring the absorption capacity of the environment to air pollutants [75]. It should be noted that in order to ensure the single nature of the indicators, the urban green area is deducted from the urban construction land area in the selection of urban construction land area data, so that the urban construction land area more effectively reflects the size of the physical space carrier for human production and life [76]. The article selects the entropy weight method to objectively assign values to each index, and assesses the amount of information obtained by determining the entropy value see Table 1 below.

The explanatory variable is the number of granted green patents. A study of a large body of literature found that patent data is a valid measure of the level of scientific and technological innovation in a certain field. The definition of green patents in this study is taken from the International Patent Green Classification List issued by the World Intellectual Property Organization, and the number of authorized green patents in each province is used as a green innovation indicator.

The mediating variable is PM2.5. Analyzed from the perspective of environmental carrying capacity, air pollutants can be divided into primary and secondary pollutants according to the formation process [76,77,78]. The so-called primary pollutants refer to pollutants emitted directly from the sources, such as carbon monoxide and sulfur dioxide. Secondary pollutants, on the other hand, are pollutants formed from primary pollutants by chemical or photochemical reactions, such as ozone, sulfate, nitrate, secondary organic particulate matter, etc. Among them, PM2.5 is the main source of primary pollutants, so this paper selects PM2.5 as a heterogeneous variable to study the impact of green innovation on environmental carrying capacity in two main aspects. On the one hand, PM2.5 has become the hot environmental issue of most concern to the society’s livelihood and the international community, whereas on the other hand, the impact of PM2.5 on economic growth, industrial structure upgrading, trade and carbon reduction are also behaviors worthy of human reflection.

The control variables include: fiscal expenditure (pe), measured by the number of fiscal expenditures within the general budget of the local government, which can represent the public services provided by the government, including haze control; industrial structure (sec), measured by the share of secondary industry output in GDP; and degree of openness to the outside world (FDI), measured by the actual amount of foreign direct investment (FDI) and the “pollution halo” and “pollution paradise” hypothesis. The former hypothesis suggests that foreign investors introduce environmentally friendly enterprises into the investment location and thus improve the host country’s environment, while the latter hypothesis suggests that foreign investors aggravate environmental pollution in the host country by transferring highly polluting enterprises. Energy saving (es) is measured by the total annual LPG supply, and the burning of fossil fuels is regarded as an important source of haze pollution, and the use of LPG reduces the burning of fossil fuels, thus contributing to haze control; population concentration (pop) is expressed by the number of people per unit density.

The data for the above-mentioned mediating and control variables are obtained from the China Statistical Yearbook (2011–2021) as well as from the official statistics publicly available on the websites of the provincial-level regional statistical bureaus.

## 3. Analysis of the Results

### 3.1. Spatial Measurement Benchmark Regression Results

The spatial econometric model cannot be established without the spatial weight matrix, which reflects the way of influence between geographical elements. In order to fully consider the reality of economic attributes of each region, this paper constructs the inverse economic distance matrix to reflect the spatial economic relationship between urban units. As the first step of spatial econometric model, a spatial autocorrelation test can analyze the distribution characteristics of corresponding variables in a geographic space, and the Moran index is generally used to reflect spatial autocorrelation in empirical studies. In this paper, the global Moran index is used to reflect the overall clustering of the environmental carrying capacity of spatial units (Table 2).

As can be seen from Table 2, the Moran indices of the environmental carrying capacity of provincial regions in China from 2010 to 2020 are all greater than zero and significantly positive at the 1% significance level, indicating that the spatial spillover of the studied environmental carrying capacity is strong, and the high values of the environmental carrying capacity are clustered with the high values and the low values are adjacent to the low values, with positive spatial correlation. Combined with the interpretation of spatial econometric model selection above, the following four non-spatial general panel models are constructed in this paper, and the LM test and Robust-LM test are used to determine whether the spatial models can be constructed. It can be found through Table 3 that the model rejects the non-spatial panel model by LM test and significance test, and the panel model with spatial factors should be selected.

Further analysis of the test results in Table 3 shows that the LM-lag, LM-error and Robust LM-error values of the model pass the test at the 1% significance level, indicating that the SEM model can be chosen. In this case, the applicability of the SDM model needs to be tested to see if it can be weakened to the SEM model by Wald and LR. The results in Table 4 prove that the test is still significant and thus the SDM model cannot be degraded into the SEM model. Meanwhile, the Hausman test of the spatial Durbin model rejects the random effects, so the SDM model with spatial fixed effects is chosen.

For the dynamic spatial Durbin model, the model parameters are consistently estimated using the great likelihood estimation method (QML) in this paper. Meanwhile, in order to compare the rationality of introducing one period of environmental carrying capacity lag, the estimation results of both the static SDM model with spatial fixed effects and the dynamic SDM model are presented in Table 5. Compared with the static spatial Durbin model, the dynamic spatial Durbin model includes one period lag of environmental carrying capacity and the coefficient is significantly positive, indicating that if the environmental carrying capacity of the previous period increases, the environmental carrying capacity of the current period will also increase, which has an obvious trend of homothetic effect and there is a path-dependent effect in time dimension. Thus, if the time lag effect of the explanatory variables is not considered, the model estimation results may be biased.

The positive regional spillover effect of green innovation is obvious, and environmental policies have different effects on green innovation in different regions. The results indicate that the spatial pattern of green innovation and spatial governance all produce significant differential results on the environment, and there is a spatial spillover effect of green technology on the enhancement of environmental carrying capacity. Since the spatial Durbin model contains both the spatial lagged terms of the explanatory and explanatory variables, Anselin and Gallo argue that the model estimation results at this point are biased for analyzing the marginal impact effects of the explanatory variables on the explanatory variables, so the results in Table 4 can only be used as a preliminary judgment. Lesage and Pace also point out that the spatial Durbin model estimation has the same problem, and find that the partial differential approach can remedy this deficiency. In the case of this paper, the effect of decomposition by this method can measure the effect of green innovation on environmental carrying capacity relatively correctly. In addition, the dynamic spatial Durbin model used in this paper includes the time lag term of environmental carrying capacity, and the effects of each explanatory variable on environmental carrying capacity need to consider the long-term effects that include the time lag factor, that is, the model estimation has short-term effects and long-term effects, which reflect the short-term and long-term effects of green innovation on environmental carrying capacity, respectively. From the data in the table, the long-term effect of green innovation on environmental carrying capacity is significantly larger than the short-term effect. The main reason is that green innovation is a long-term behavior, while environmental carrying capacity is also a long-term behavior. Therefore, there is a difference between short-term and long-term effects of green innovation on environmental carrying capacity. In summary, the results of further effect decomposition are shown in Table 6. From the total effect of Table 6, it is clear that green innovation has a significant contribution to the improvement of environmental carrying capacity in both the short and long term.

### 3.2. Robustness Test

#### 3.2.1. Transformation Space Weight Matrix

The above empirical results are analyzed under the inverse economic distance matrix dominated by economic distance. In this paper, we use the nested weight matrix of economic-cum-geospatial distance for robustness testing, and comparing the results in Table 6 and Table 7, the signs are basically consistent with the significance and the underlying regression model, indicating that the effect of green innovation on environmental carrying capacity is robust.

#### 3.2.2. Analysis of Endogeneity Problem

Although the dynamic spatial Durbin model can solve the endogeneity problem caused by omitted variables, the endogeneity problem that green innovation and environmental carrying capacity are mutually causal cannot be solved. Based on the use of the dynamic spatial Durbin model, this paper further takes the explanatory variables and their spatial lagged terms of second and third order, green innovation and their respective spatial lagged terms of second and third order as the instrumental variables through the systematic GMM method. In addition, the air circulation coefficient is used as one of the instrumental variables. As an exogenous variable formed and objectively existing in natural geography, the air circulation coefficient has an impact on the environmental carrying capacity, and there is a correlation between it and green innovation, which is a more appropriate instrumental variable.

As shown in Table 8, the Sargan test accepts the original hypothesis at the 10% significance level, so the instrumental variables selected above are valid; in addition, the AR (1) test is significant and the AR (2) test is insignificant, indicating that the nuisance terms are not autocorrelated, which shows that the estimation results of this paper using the systematic GMM approach to address endogeneity are reasonable. Table 4, when compared with the estimated results in Table 8, even though the sign of the green innovation coefficient changes, neither of them is significant, indicating the robustness of the underlying regression results. Meanwhile, both the lagged term and spatial lagged term of environmental carrying capacity are significantly positive, which verifies the existence of path dependence in the time dimension and significant spatial demonstration effect of environmental carrying capacity.

### 3.3. Mediating Effect Test

Environmental pollution PM2.5 was selected as the mediating variable of green innovation affecting environmental carrying capacity. Drawing on Feng Han and Ligao Yang’s mediating effect test, the significance of the coefficients is used to determine the role of PM2.5 in the relationship between green innovation and environmental carrying capacity by constructing a recursive model.

The estimation results of Equation (1) in Table 9 are consistent with the results of the decomposition of the effects of the base regression in the previous section and will not be repeated here. The results of Equation (2) to test the effect of green innovation on reducing PM2.5 concentration show that the effect of green innovation is significant, and the total effect of PM2.5 in Equation (3) is significant and has a significant inhibitory effect on the improvement of environmental carrying capacity of the surrounding urban provinces in the short term, where the short-term direct effect is −0.011 and the short-term indirect effect is −0.125 and the absolute value of the short-term indirect effect is greater than the absolute value of the short-term direct. The absolute value of the short-term indirect effect is greater than the absolute value of the short-term direct effect, and the long-term direct effect is greater than the long-term indirect effect, but it is not significant. In summary, PM2.5 plays a part of the mediating effect in the process of green innovation affecting environmental carrying capacity, indicating that PM2.5 plays an important role in the transmission mechanism of green innovation affecting environmental carrying capacity. That is, it shows that in areas with higher PM2.5 concentration or poorer environmental quality, green innovation has a weaker ability to enhance environmental carrying capacity; in areas with lower PM2.5 concentration or higher environmental quality, green innovation has a stronger ability to enhance environmental carrying capacity. Compared to areas with higher PM2.5 concentration or poorer environmental quality, green innovation on environmental carrying capacity is influenced by the local environmental quality. Therefore, the environmental carrying capacity of green innovation is stronger for areas with lower PM2.5 concentration or higher environmental quality.

## 4. Conclusions and Recommendations

### 4.1. Conclusions

This paper selects the 2010–2020 provincial-level regions of China as samples, adopts a multi-indicator comprehensive evaluation method to comprehensively, objectively and scientifically evaluate the environmental carrying capacity of air pollution from two dimensions: natural resource endowment and human activity impact, and also measures the green innovation of each province, city and autonomous region to explore the specific impact of green innovation on environmental carrying capacity and its spatial spillover effect; it also explores the heterogeneous impact of green innovation on environmental carrying capacity under different pollution environments.

(1) Whether in the short term or in the long term, green innovation makes a significant contribution to the improvement of environmental carrying capacity; green innovation can bring beneficial environmental effects not only to reduce the increase of pollutants, but also to purify and absorb the pollutants already produced from the direction of pollution treatment and so on. Due to the existence of environmental carrying capacity, pollution is not absolutely irreversible; the environment has the possibility of repair and treatment, and the environmental carrying capacity directly affects the whole process of pollutant generation. Environmental carrying capacity will be enhanced to reduce the concentration of pollutants once the pollutants exceed the environmental carrying capacity form cumulative pollution, causing serious damage to the ecology; the reduction of the concentration of pollutants will also generate the environmental carrying capacity of sustainable maintenance, and the two form a dynamic virtuous cycle. It can be seen that there is a significant and complex correlation between pollutant concentration and environmental carrying capacity, and it can be speculated that the sensitivity of environmental restoration capacity under different pollutant concentrations is affected by various factors; for example, the effect of green innovation on environmental carrying capacity under different pollutant levels may also be affected by different pollutants.
(2) Green innovation has a significant spatial spillover effect on the enhancement of environmental carrying capacity. This indicates that green innovation does not always play a positive role in enhancing the environmental carrying capacity, and in some cases this enhancement will be weakened. This indicates that ecological protection and environmental management is a complex system project, which cannot rely on a single element or a complete market mechanism to get the maximum benefit. The development of green innovation requires the establishment of sound environmental protection rules and regulations and supporting regulations, targeted protection and incentives for relevant green innovation and coordinated development between regions and sectors to maximize the benefits of green water and green mountains.
(3) In the process of green innovation affecting environmental carrying capacity, PM2.5 plays a part in the mediating effect, indicating that PM2.5 plays an important role in the transmission mechanism of green innovation affecting environmental carrying capacity. This shows that the two-way influence relationship between pollutants and environmental carrying capacity affects the extent of green innovation in pollution control, in which the regional environment is in a sustainable state with low PM2.5 concentration and green innovation can more effectively promote the environmental carrying capacity to improve the ability to clean pollutants, while the region with high PM2.5 concentration and severe pollution is closer to the development from emergency critical scenario to pessimistic scenario. This conclusion demonstrates that the pollution level represented by PM2.5 concentration is an important regulating variable for green innovation to improve the environmental carrying capacity, and also provides ideas for optimizing air pollution management.

### 4.2. Recommendations

Green innovation is essentially ecotechnological innovation, with the development of green industries as an important initiative to promote economic restructuring and highlight the concept and connotation of green on a macro level, and the promotion of energy conservation and efficiency in the production, distribution, distribution, consumption and construction of enterprises on a micro level. The significance of the research in this paper proposes the profound implication of green innovation on environmental carrying capacity enhancement, pointing out that green innovation has a positive contribution to environmental carrying capacity, and in this sense, China needs to further promote eco-technological innovation, advance a mode of economic growth and social development that aims at efficiency, harmony and sustainability, promote an accelerated transformation of the mode of economic development, actively cultivate new economic featuring low carbon emissions growth, reduce consumption, reduce losses and pollutant emissions, stop waste through green innovation in energy conservation and emission reduction, reduce consumption, reduce losses and pollutant emissions, stop waste in all aspects of energy production to consumption, use energy effectively and rationally, practice the concept of green development, build a resource-saving and environment-friendly society, continuously promote the modernization of China’s green innovation governance system and governance capacity, continuously promote regional environmental carrying capacity, and in the process of economic growth focus on environmental, social and ecological effects to build a beautiful China.

## Figures and Tables

**Table 1:** Environmental Carrying Capacity (ECC) Indicator System.

Secondary Indicators	Specific Indicators	Unit	Nature	Average Weight
Natural Conditions Endowment	Surface water resources	billion m^3^	Positive	0.225
	Wetland area	million hm^2^	Positive	0.216
	Forest area	million hm^2^	Positive	0.168
	Annual precipitation	mm	Positive	0.142
Human Activity Impacts	Greening coverage of built-up areas	%	Positive	0.078
	Urban construction land area	km^2^	Negative	0.076
	Environmental emergencies	times	Negative	0.095

**Table 2:** Regional Environmental Carrying Capacity Moran’s I Statistical Values 2010–2020.

Year	*Moran’s I* Values	Year	*Moran’s I* Values
2010	0.056 ***	2016	0.048 ***
2011	0.048 ***	2017	0.064 ***
2012	0.052 ***	2018	0.071 ***
2013	0.061 ***	2019	0.062 ***
2014	0.055 ***	2020	0.072 ***
2015	0.061 ***	Average	0.058 ***

Note: *** indicates significant at the 1% level.

**Table 3:** LM test for the ordinary panel model.

Inspection	Hybrid OLS	Space Fixation	Fixed Time	Double Fixed in Time and Space
*LM-lag*	236.523 ***	231.821 ***	202.132 ***	202.085 ***
*Robust LM-lag*	9.236 ***	9.123 ***	1.852	1.812
*LM-error*	326.785 ***	326.782 ***	252.023 ***	251.233 ***
*Robust LM-error*	102.356 ***	103.758 ***	61.783 ***	61.247 ***

Note: *** indicates significant at the 1% level.

**Table 4:** Wald test and LR test for spatial Durbin model.

*Wald lag*	71.256 *** (0.000)
*LR lag*	76.425 *** (0.000)
*Wald error*	19.485 ** (0.013)
*LR error*	20.126 ** (0.011)

Note: ***, ** indicates significant at the 1% and 5% level.

**Table 5:** Spatial measurement benchmark regression results.

Variables	OLS Returns	Static SDM Model	Dynamic SDM Model
*L.ECC*			0.925 ***
*Gino*	0.825 ***	0.752 ***	0.142
*pe*	−0.253	−0.225	−0.208
*sec*	−0.335 *	−0.305 *	−0.204 *
*FDI*	1.126	1.058	0.523
*es*	−5.652 **	−5.456 **	−0.032
*pop*	−3.325	−3.365	−0.192
*wxGino*		1.085 ***	0.589 ***
*wxpe*		−0.563 **	−1.389 ***
*wxsec*		−0.652 ***	−0.488 ***
*wxFDI*		2.112 *	1.563 *
*wxes*		−14.059 ***	−1.752
*wxpop*		−36.256 ***	−4.562
*ρ*		0.189 ***	0.098 ***
*R* ^2^	0.352	0.198	0.752
log-L		−5510.563	−3852.23

Note: ***, **, * indicate significant at the 1%, 5% and 10% levels.

**Table 6:** Effect decomposition of the base regression.

Variables	Short-Term			Long-Term		
	Direct	Indirect	Total	Direct	Indirect	Total
	Effect	Effects	Effect	Effect	Effects	Effect
*Gino*	0.156	0.698 ***	0.865 ***	6.895	1.023	7.856 ***
Control variables	Control	Control	Control	Control	Control	Control

Note: *** indicates significant at the 1% level.

**Table 7:** Decomposition of spatial effects under nested matrices.

Variables	Short-Term			Long-Term		
	Direct	Indirect	Total	Direct	Indirect	Total
	Effect	Effects	Effect	Effect	Effects	Effect
*Gino*	0.002	2.459 **	2.452 **	0.562	2.956	3.126 **
Control variables	Control	Control	Control	Control	Control	Control

Note: ** indicates significant at the 5% level.

**Table 8:** System GMM Estimation Results.

*Gino*	0.3543	1.273	*WxGino*	1.039	2.593
*L.ECC*	0.746	365.341	*wxL.ECC*	0.152	36.926
*Sargan-test*	246.583	0.265			

**Table 9:** Mediating effect test based on PM2.5.

Variables	Type of Effect	Equation (1)	Equation (2)	Equation (3)
*InPM2.5*	Short-term direct effects			−0.011
	Short-term indirect effects			−0.125 ***
	Short-term aggregate effect			−0.142
	Long-term direct effects			0.852
	Long-term indirect effects			0.048
	Total long-term effect			0.856 ***
*Gino*	Short-term direct effects	0.152 ***	0.054 **	0.138
	Short-term indirect effects	0.623 ***	0.421	0.652 ***
	Short-term aggregate effect	0.852	0.489	0.754 ***
	Long-term direct effects	7.112	0.174	−6.865
	Long-term indirect effects	1.103	1.121	2.412
	Total long-term effect	8.132 ***	1.125 ***	−5.568 ***
Control variables	Control	Control	Control	Control

Note: ***, ** indicate significant at the 1% and 5% levels.

## Data Availability

No new data were created or analyzed in this study. Data sharing is not applicable to this article.
